# Supplementary material for: A Clinically Aligned Murine Model of Electroconvulsive Stimulation Reverses Social Aversion and Displays Fear Memory Impairment After Chronic Social Defeat Stress
Source: bioRxiv. 2025 Feb 14:2025.02.12.637719. Preprint. [Version 1] doi: 10.1101/2025.02.12.637719 (PMC11844563; doi:10.1101/2025.02.12.637719)
Supplement: Supplement 1 [file media-1.pdf]

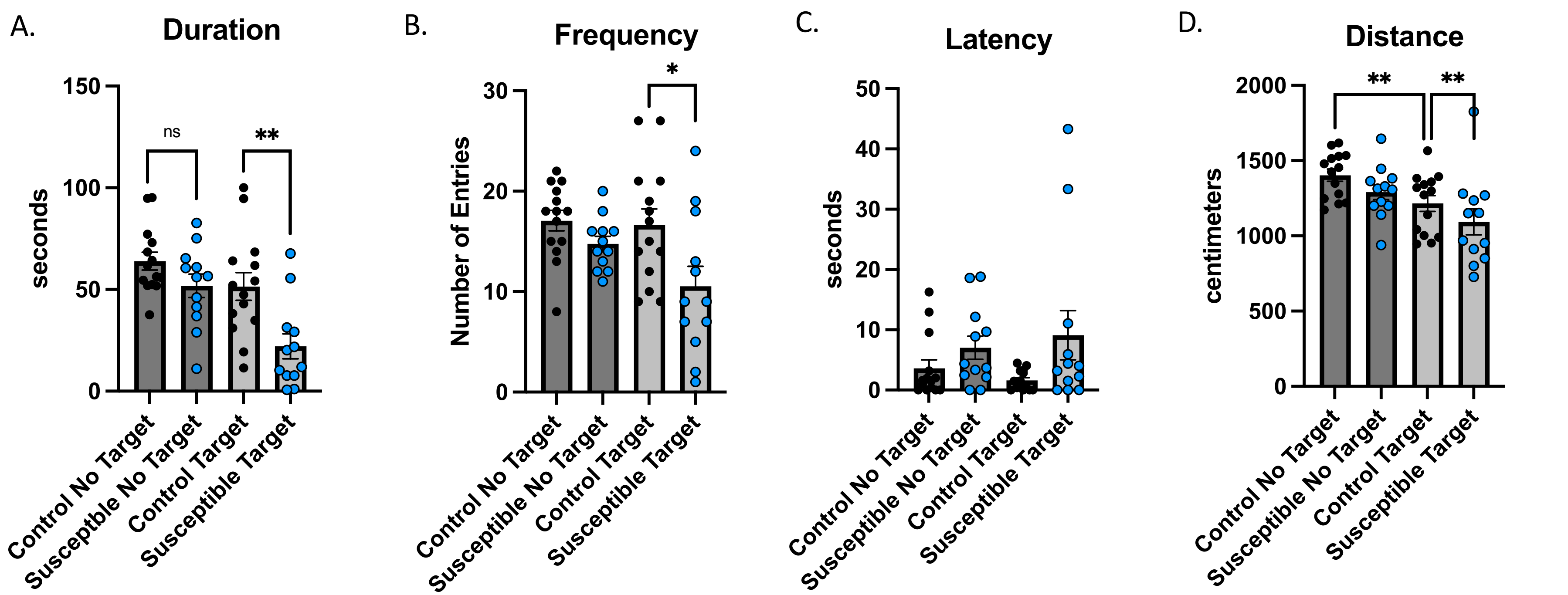

Supplemental Figure 1. Social interaction test details for susceptible and control mice. **(A)** The presence of a social target (CD1 mouse) decreased the amount of time susceptible mice (n=12) spent in the interaction zone compared to control mice (n=14, *P*=0.0041). The absence of a social target did not influence the time spent in the interaction zone. **(B)** The presence of a social target decreased the frequency of entries to the interaction zone in susceptible mice (n=12) compared to control mice (n=14) [ANOVA: group by target interaction F (3,48) = 4.32, P = 0.0089. Post-hoc students t-test between groups with the social target was significant (P=0.0235), while there was no difference without the social target (P=0.0874). **(C)** The presence of a social target did not change the time for the first entry latency into the interaction zone in susceptible mice (n=12) compared to control mice (n=14). [ANOVA: group by target interaction F (3, 48) = 2.253, P = 0.0942. **(D)** Susceptible mice and control mice showed differences in distance traveled during the social interaction test [ANOVA: group by target interaction F(3,48) = 4.931, P = 0.0046. Post-hoc student's t-tests showed no difference between groups under conditions without a target (P = 0.0952) but a significant difference between groups under conditions with a target (P = 0.0024)] as well as a significant decrease in control mice with the social target present (P=0.0085).

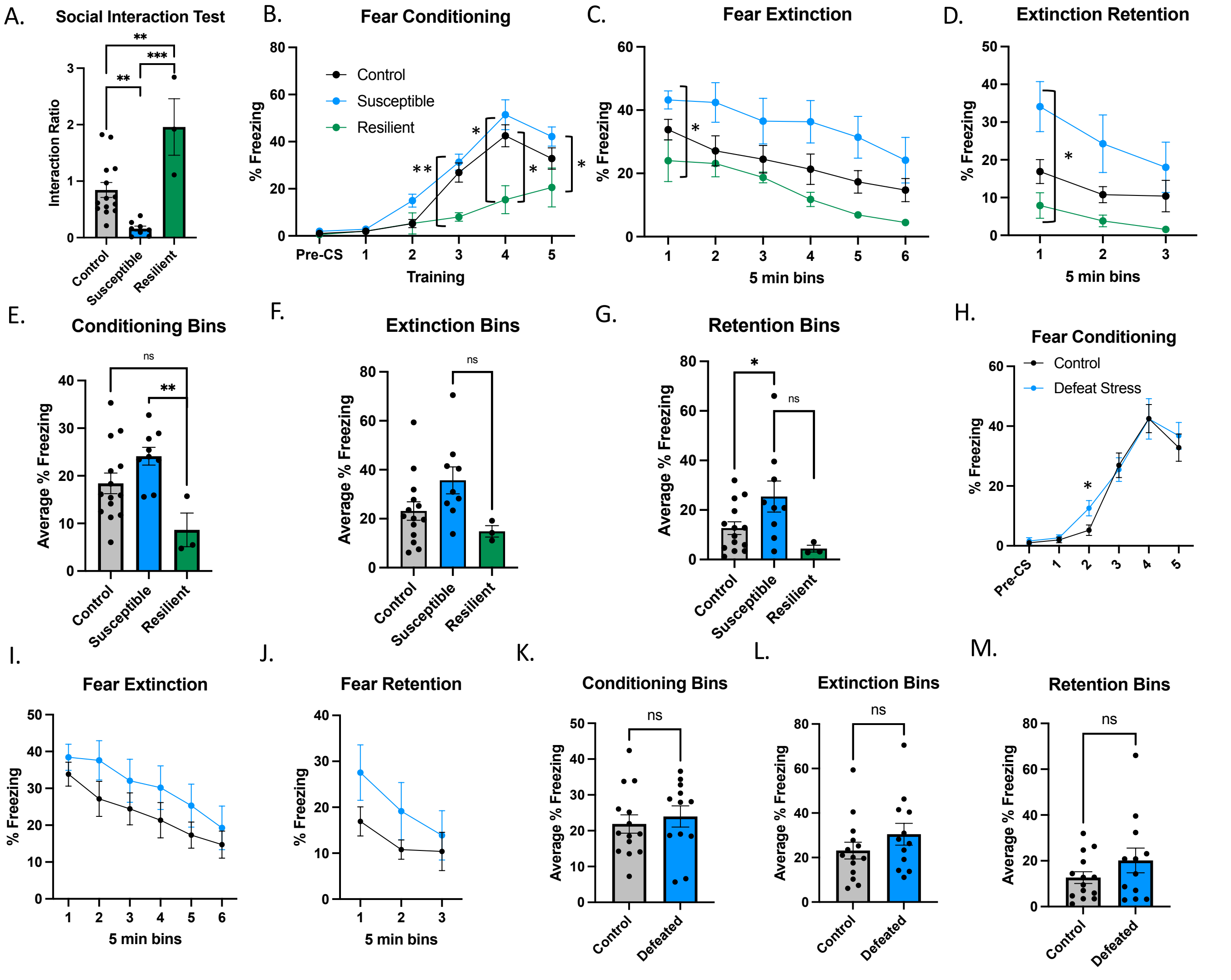

Supplemental Figure 2. Fear memory acquisition and extinction after chronic social defeat stress. **(A)** Mice displayed a resilient (n=3) phenotype defined by a SIT >1, while susceptible mice (n=9) and unstressed controls (n=14) [ANOVA: group by trials interaction F(13,9)=8.6, P=0.0014], post-hoc pairwise comparisons revealed significant differences between control and susceptible (P=0.0011), susceptible and resilient mice (P=0.0001) and between control and resilient mice (P=0.0066). **(B)** Use of tone (US) and shock (CS) for CS-US pairings in the acquisition of fear memory. Percent freezing during fear conditioning over six five-minute bins [ANOVA: group by bins interaction F(10,115)=2.609, P=0.0068], post-hoc unpaired comparisons showed a significant difference at timepoint 2 between control and susceptible (P=0.00489) and timepoints 3-5 between susceptible and resilient (P=0.00373, 0.01202, 0.02769, respectively) and at timepoint 4 between control and resilient mice (P=0.02283). **(C)** Fear extinction over 30 minutes in six bins [ANOVA: group by bins interaction F(5,138)=3.562, P=0.0046); post-hoc students t-test showed significant differences between susceptible and resilient mice at timepoint one (P=0.0107), and no significant differences between susceptible and control or between control and resilient mice. **(D)** Retention of fear memories after extinction (extinction retention) had no interaction between the three groups [ANOVA: group by trials interaction F(4,69) = 0.2919, P=0.8823]; post-hoc student t-tests had one difference between control and susceptible mice at the first timepoint (P=0.0169); there were no significant differences between control and resilient mice or susceptible and resilient mice. **(E)** Fear conditioning bins of control, susceptible and resilient averaged [ANOVA: group by bins interaction F(2,23)=5.462, P=0.0115); post-hoc students t-test showed a significant difference between susceptible and resilient mice (P=0.0024). **(F)** There were no differences in fear extinction between all three groups [ANOVA: group by bins interaction F (2, 23) = 3.194, P=0.0597. **(G)** Retention of fear memories after extinction between the three groups [ANOVA: group by bins interaction F(2,23)=3.850, P=0.0361; post-hoc students t-tests showed a significant difference between control and susceptible mice (P=0.0433). **(H-M)** Comparisons between control mice and all mice that underwent defeat stress (defeated = susceptible + resilient). **(H)** Control and defeated mice show no difference over CS-US pairings in the acquisition of fear memory [ANOVA: trials by group interaction F(5,66)=0.6604, P=0.6547. Post-hoc test showed no difference between groups under presentations of CS-US pairings except in trial 2, (P=0.0238)]. **(I)** 24 hours after fear conditioning, control mice (n=14) and defeated mice (n=12) underwent a series of fear extinction trials where the presentation of the tone (US) did not elicit a shock (CS). A group by trials repeated measured ANOVA revealed no significant differences, F(5,144)=0.9868. **(J)** 48 hours after fear extinction, control mice (n=14) and defeated mice (n=12) underwent a series of fear extinction trials where the presentation of the tone (US) did not elicit a shock (CS) to assess the retention of fear memories. A group by trials repeated measured ANOVA revealed no significant differences (F(2,72), P=0.7303). **(K)** Fear conditioning bins of control and defeated mice were not significantly different with a students t-test (P=0.5971). **(L)** There were no differences in fear extinction between control and defeated mice (P=0.2403). **(M)** Retention of fear memories after extinction between control and defeated mice revealed no significant differences (P=0.2013).

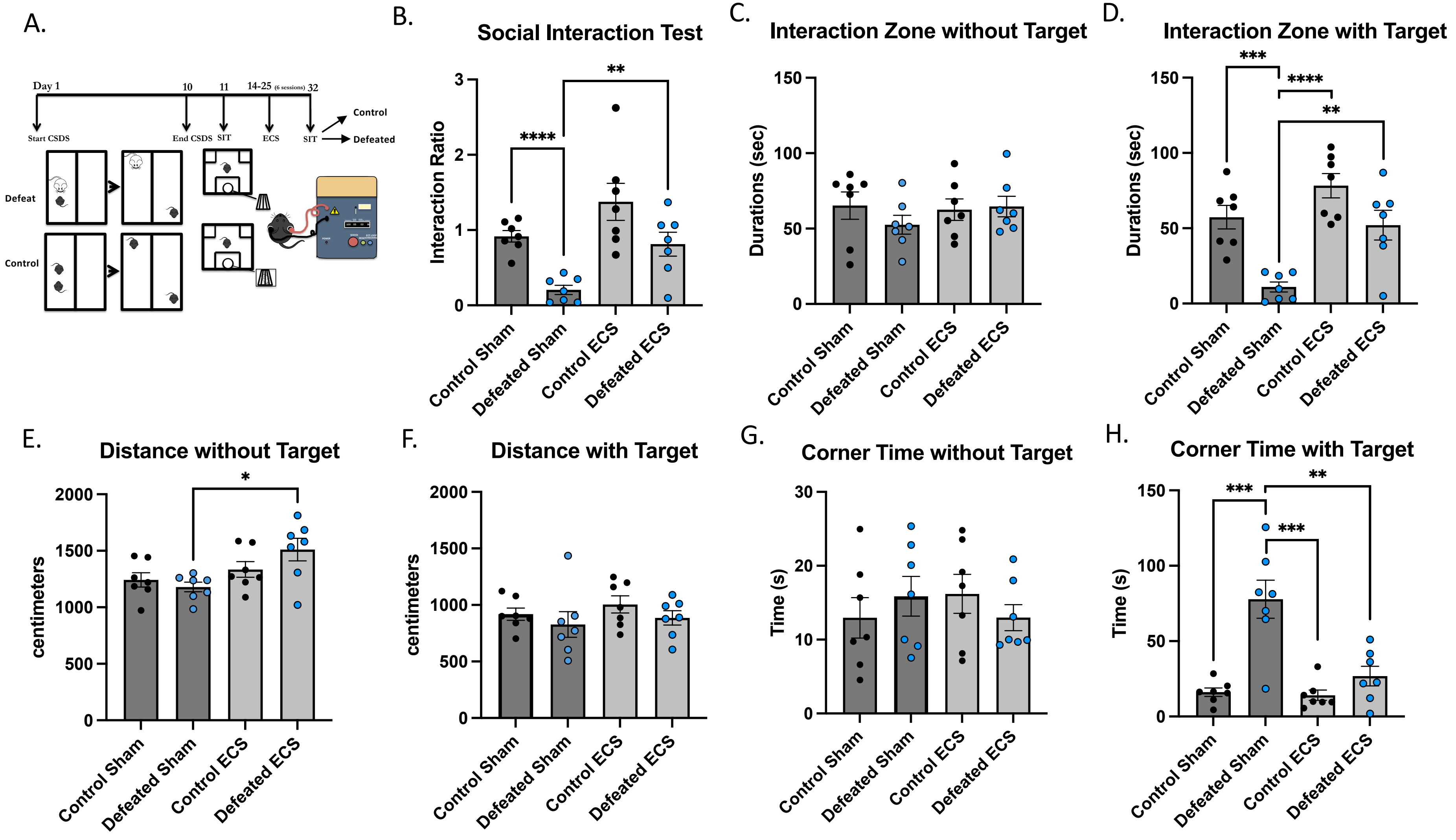
A.
Day 1
10
11
14-25 (6 sessions) 32
Start CSDS
End CSDS
SIT
ECS
SIT
Control
Defeated
Defeat
Control
B.
Social Interaction Test
Interaction Ratio
****
**
Control Sham
Defeated Sham
Control ECS
Defeated ECS
C.
Interaction Zone without Target
Durations (sec)
D.
Interaction Zone with Target
Durations (sec)
***
****
**
E.
Distance without Target
centimeters
*
F.
Distance with Target
centimeters
G.
Corner Time without Target
Time (s)
H.
Corner Time with Target
Time (s)
***
**
***

Supplemental Figure 3. Reversal of social aversion from chronic social defeat stress after electroconvulsive stimulation with resilient mice included. **(A)** Diagram of the experimental design. **(B)** The presence of a social target decreased the social interaction test of defeated sham mice (n=7) compared to control sham mice (n=7), ANOVA: group by treatment interaction $F(3,24)=9.683$, $P=0.0002$. Post-hoc unpaired students t-test revealed significant differences between control and defeated sham mice ($P<0.0001$), between defeated sham and defeated ECS ($P=0.0039$), but not between control sham and ECS (n=7), $P=0.1015$, or control sham and defeated ECS ($P=0.5642$), or control ECS (n=7) and defeated ECS ($P=0.0790$). **(C)** There was no significant interaction of time spent in the interaction zone between groups when the social target was absent (ANOVA: $F(3,24)=0.6428$, $P=0.5959$), but there were **(D)** significant differences between groups when the social target was present (ANOVA: $F(3,24)=13.47$, $P<0.0001$). Post-hoc unpaired student's t-test revealed significances between defeated and control sham ($P=0.0002$), control ECS and defeated sham ($P<0.0001$), defeated ECS and sham ($P=0.0020$), but not between control ECS and defeated ECS ($P=0.0622$) or control sham and defeated ECS ($P=0.6815$). **(E)** There was significant group by treatment interaction in locomotion between the groups ANOVA: F(3,24)=4.006, $P=0.0191$. A post-hoc unpaired students t-test showed a significant difference between defeated sham and ECS ($P=0.0103$). **(F)** There was not an interaction when the social target was present ANOVA: group by treatment $F(3,24)=0.8572$, $P=0.4767$. **(G)** There were no significant group by treatment interaction in the time spent in the corners of the arena when the social target is not present (ANOVA: F(3,24)=0.5068, $P=0.6813$), but there were when the social target was present **(H)** ANOVA: F(3,24)=16.20, $P<0.0001$. Post-hoc unpaired student's tests revealed significant differences between sham control and defeated mice ($P=0.0005$), defeated sham and control ECS ($P=0.0004$) and defeated sham and ECS ($P=0.0037$), but not between susceptible and control ECS mice ($P=0.1094$).

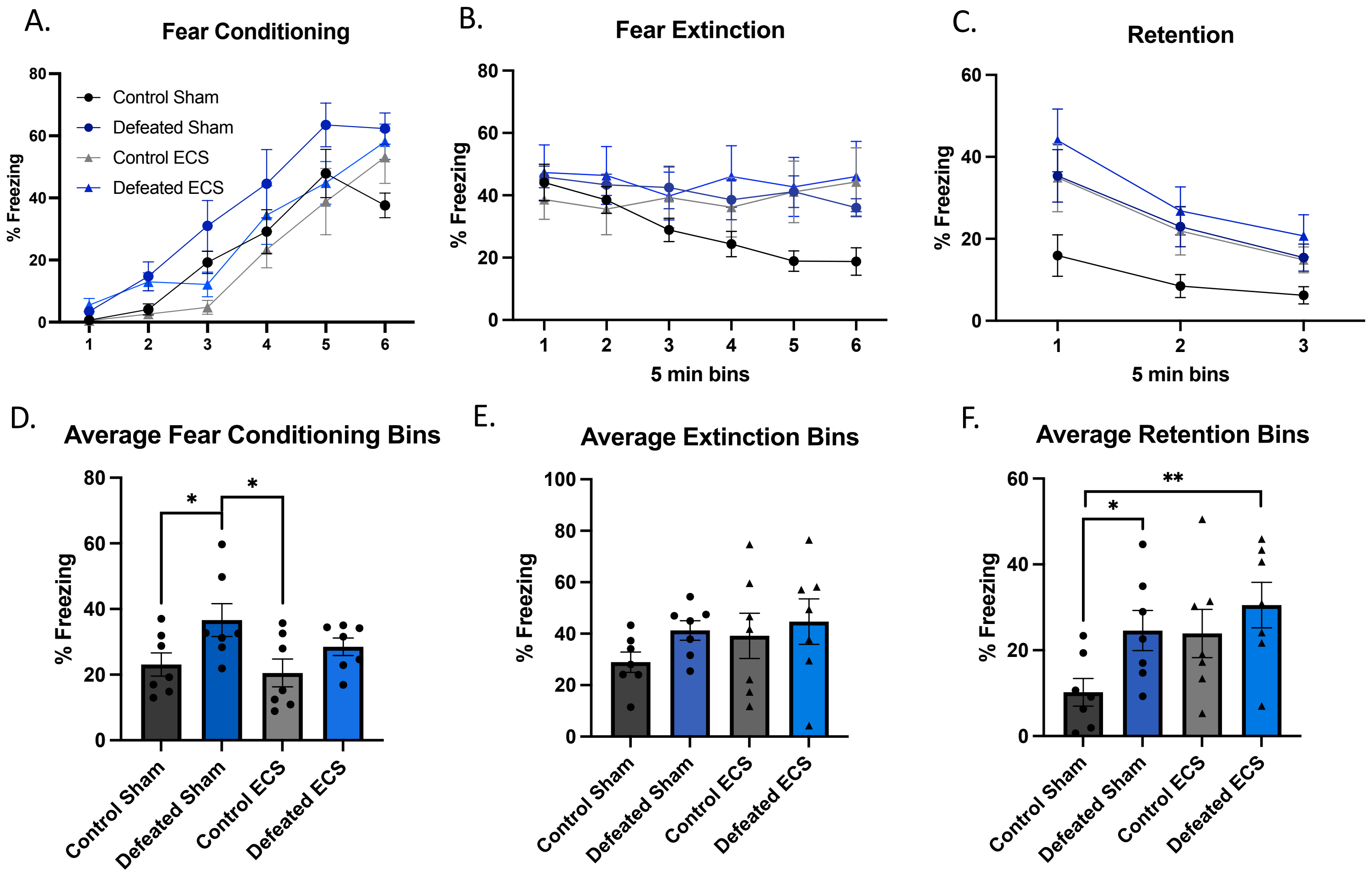

Supplemental Figure 4. Electroconvulsive stimulation of mice after chronic social defeat stress enhances fear acquisition and retention. **(A)** Use of tone (US) and shock (CS) for CS-US pairings in the acquisition of fear memory in control mice or defeated mice and then either ECS or sham ECS. Percent freezing during fear conditioning over six five-minute bins showed no groups by trial interaction, ANOVA: $F(15,144)=0.9965$, $P=0.4621$. **(B)** Fear extinction over 30 minutes in six bins [ANOVA: group by bins interaction $F(5,144)=0.7127$, $P=0.7687$) **(C)** Retention of fear memories after extinction (extinction retention) had no group by trials interaction between control and susceptible mice, ANOVA: $F(2,63)=0.6173$, $P=0.7157$. **(D)** Averaged fear conditioning bins of control and defeated mice showed a significant group by treatment interaction ANOVA: $F(3,24)=3.232$, $P=0.0401$. Post-hoc unpaired students t-tests revealed significant differences between control and defeated sham ($P=0.0476$) and between defeated sham and control ECS ($P=0.0302$), but not between control and defeated ECS (P=0.1363), defeated ECS and control sham ($P=0.2453$) and defeated sham and defeated ECS ($P=0.1788$). **(E)** There were no differences in fear extinction between the groups, ANOVA: $F(3,24)=0.9922$, $P=0.4132$. **(F)** Retention of fear memories after extinction between control and susceptible mice was significantly different, ANOVA: group by treatment interaction $F(3,24)=3.214$, $P=0.0408$. Post-hoc unpaired student's t-test revealed significant differences between control and defeated sham (P=0.0264), between control sham and defeated ECS (P=0.0066), but not between control sham and control ECS (P=0.0561), control ECS and defeated sham (P=0.9262), or defeated sham and ECS (P=0.4181).

Nominal p-values < 0.05 – 873 genes

gene_id	baseMean	log2FoldChange	lfcSE	stat	pvalue	padj
Arl4d	1007.50114	1.1621863	0.11631053	9.992099	1.650492e-23	1.155179e-19
Dusp6	1641.05131	1.1276822	0.12558010	8.979785	2.712975e-19	9.494055e-16
Rgs2	3202.49318	1.4450231	0.18190684	7.943754	1.961530e-15	4.576250e-12
Egr3	3764.57729	0.6974991	0.09155288	7.618538	2.565651e-14	4.489248e-11
Dusp14	1057.26382	0.5874298	0.08412976	6.982426	2.901260e-12	4.061184e-09
Homer1	3922.49078	0.4193676	0.07165024	5.852983	4.828348e-09	4.827658e-06
Rprml	1266.20204	0.5465079	0.09315398	5.866716	4.445104e-09	4.827658e-06
Ascl1	156.55670	−0.7364989	0.13273699	−5.548558	2.880357e-08	2.519952e-05
Egr2	496.97254	0.8478173	0.15580421	5.441556	5.281721e-08	4.107418e-05
Mycn	167.11473	−0.6598825	0.12256376	−5.383994	7.285098e-08	5.098840e-05
Sgk1	1898.18247	0.7443437	0.14056435	5.295395	1.187595e-07	7.556345e-05
Fhl2	569.98997	0.4617027	0.09045690	5.104118	3.323411e-07	1.938379e-04
Nr4a1	4162.48363	0.6366453	0.12549778	5.072961	3.916734e-07	2.108709e-04
Tob1	895.35410	−0.2918316	0.05960077	−4.896440	9.758850e-07	4.878728e-04
Akap5	3774.28524	0.4125044	0.08550957	4.824073	1.406561e-06	6.563014e-04
C130074G19Rik	644.07792	−0.5981682	0.12696907	−4.711133	2.463429e-06	1.061495e-03

Nominal P-value < 0.05; Log2fold change > 1

gene_id	baseMean	log2FoldChange	lfcSE	stat	pvalue
Arl4d	1007.501142	1.162186	0.1163105	9.992099	1.650492e-23
Dusp6	1641.051311	1.127682	0.1255801	8.979785	2.712975e-19
Rgs2	3202.493179	1.445023	0.1819068	7.943754	1.961530e-15
Gm39741	14.467938	1.098928	0.3518999	3.122842	1.791141e-03
Tbata	13.920031	1.117258	0.3610578	3.094402	1.972099e-03
Gm4926	10.905821	1.089456	0.3675822	2.963844	3.038223e-03
Dct	9.524389	1.182761	0.4522182	2.615465	8.910602e-03
Gm18157	32.807271	7.328458	2.9732025	2.464837	1.370758e-02

Adjusted P-value < 0.05; Log2fold change > 1

gene_id	baseMean	log2FoldChange	lfcSE	stat	pvalue
Arl4d	1007.501	1.162186	0.1163105	9.992099	1.650492e-23
Dusp6	1641.051	1.127682	0.1255801	8.979785	2.712975e-19
Rgs2	3202.493	1.445023	0.1819068	7.943754	1.961530e-15

Supplemental Figure 5. Significant changes in bulk mRNA levels in Nacc ECS compared to Sham unstressed mice
